# Supplementary material for: Deep learning predicts cardiac output from seismocardiographic signals in heart failure
Source: medRxiv. 2025 Jul 14:2025.07.11.25331386. Preprint. [Version 1] doi: 10.1101/2025.07.11.25331386 (PMC12338910; doi:10.1101/2025.07.11.25331386)

Central Illustration. We describe a novel algorithm utilizing (1 and 2) publicly available wearable patch-derived electrocardiographic (ECG) and triaxial seismocardiographic (SCG) signals, combined with body mass index (BMI), in a (3) deep learning model to (4) predict cardiac output in heart failure.

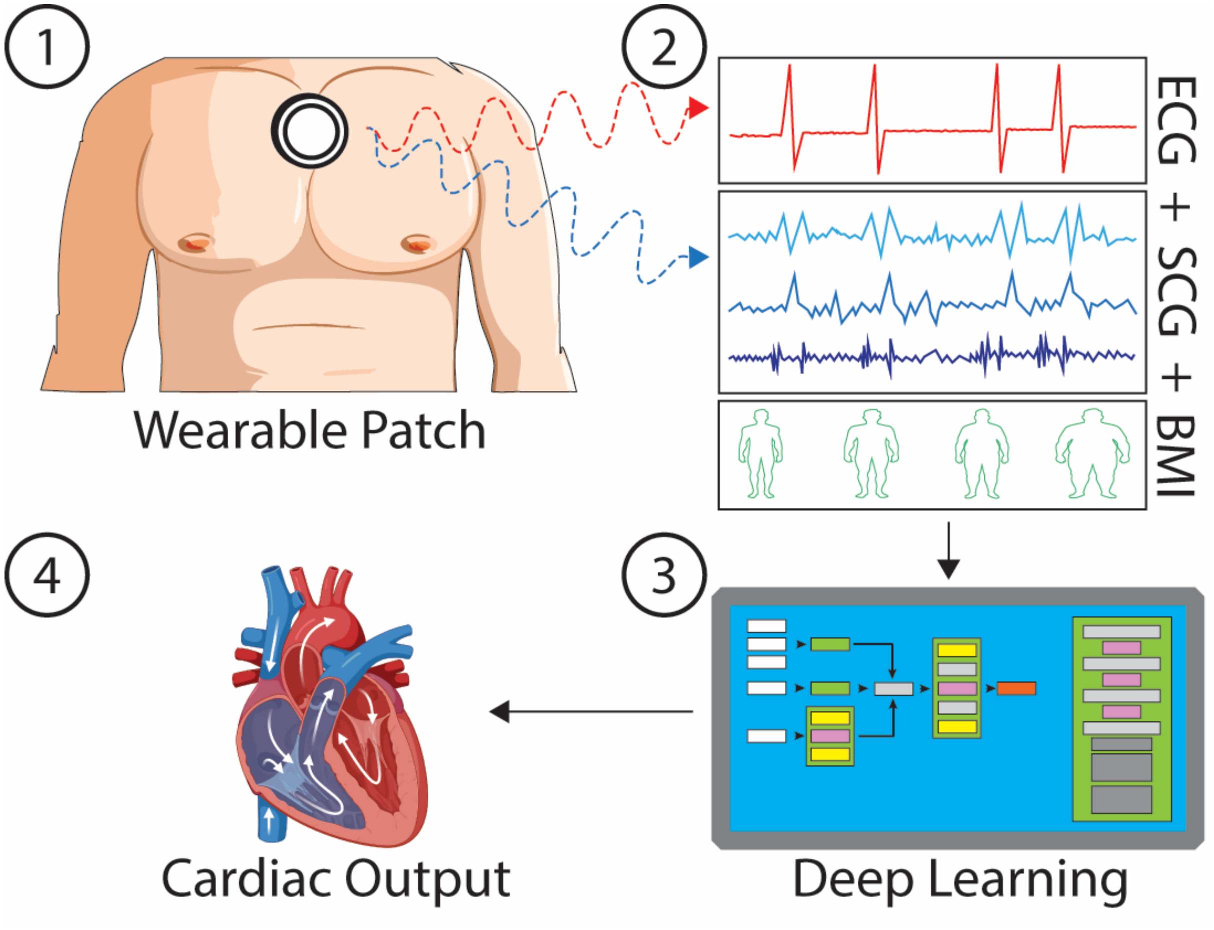

Supplement: Supplement 2 [file media-2.pdf]
